# Congenital heart diseases: population-based cross-sectional study and spatial analysis, São Paulo, 2012–2022

**DOI:** 10.1590/S2237-96222026v35e20250519.en

**Published:** 2026-03-16

**Authors:** Amarílis Bahia Bezerra, Ligia Vizeu Barrozo, Alfredo Pereira de Queiroz

**Affiliations:** 1 Universidade de São Paulo, Departamento de Geografia, São Paulo, SP, Brazil

**Keywords:** Heart Defects, Congenital, Spatial Analysis, Maternal and Child Health, Epidemiology, Cross-Sectional Studies, Cardiopatías Congénitas, Análisis Espacial, Salud Materno-Infantil, Epidemiología, Estudios Transversales

## Abstract

**Objectives:**

To identify spatial clusters of risk and analyze maternal sociodemographic and obstetric factors associated with the occurrence of congenital heart diseases in the municipality of São Paulo between 2012 and 2022.

**Method:**

Population-based cross-sectional study and ecological spatial analysis using data from the Live Birth Information System (*Sistema de Informações sobre Nascidos Vivos*, SINASC) from 2012 to 2022 in the municipality of São Paulo. The outcomes were newborns diagnosed with congenital heart disease (International Statistical Classification of Diseases and Related Health Problems 10th Revision: Q20–Q28) and, as the comparison group, all other live births. Spatial scan analysis and logistic regression were used to estimate *odds ratio* (OR) and 95% confidence intervals (95%CI).

**Results:**

A total of 1,777,316 live births were recorded, of which 9,293 presented congenital heart diseases. Spatial analysis identified a cluster of cases in the municipality’s northern area. Occurrence was associated with: residence in areas of spatial clustering (OR 2.97; 95%CI 2.82; 3.12), preterm pregnancy (OR 2.61; 95%CI 2.45; 2.78), triple or higher-order pregnancy (OR 2.60; 95%CI 1.57; 4.31), twin pregnancies (OR 1.14; 95%CI 1.00; 1.30), stable union (OR 1.44; 95%CI 1.37; 1.53), level of education between 1 to 7 years (OR 1.26; 95%CI 1.14; 1.39), 8 to 11 years (OR 1.20; 95%CI 1.11; 1.29), age between 35 to 49 years (OR 1.19; 95%CI 1.11; 1.28), and Asian (OR 1.81; 95%CI 1.47; 2.24) and Black race/skin color (OR 1.16; 95%CI 1.06; 1.27).

**Conclusion:**

Individual maternal characteristics and territorial factors were associated with the occurrence of congenital heart diseases, revealing population profiles and priority areas for health actions.

## Resumen

**Objetivos:**

Identificar agrupamientos espaciales de riesgo y analizar los factores sociodemográficos y obstétricos maternos asociados a la ocurrencia de cardiopatías congénitas en el municipio de São Paulo entre 2012 y 2022.

**Método:**

Estudio transversal de base poblacional y análisis espacial de carácter ecológico, utilizando datos del Sistema de Información sobre Nacidos Vivos, de 2012 a 2022, en el municipio de São Paulo. Los desenlaces fueron los recién nacidos con diagnóstico de cardiopatía congénita (10.ª Revisión de la Clasificación Internacional de Enfermedades: Q20 a Q28) y el grupo de comparación, los demás nacidos vivos. Se aplicaron análisis de escaneo espacial y regresión logística para estimar razones de probabilidades (*odds ratio*, OR) e intervalos de confianza del 95% (IC95%).

**Resultados:**

Se registraron 1.777.316 nacidos vivos, de los cuales 9.293 presentaron cardiopatías congénitas. El análisis espacial identificó un agrupamiento de casos en la Zona Norte del municipio. La ocurrencia se asoció con: residencia en áreas de agrupamiento espacial (OR 2,97; IC95% 2,82; 3,12), embarazos pretérmino (OR 2,61; IC95% 2,45; 2,78), embarazo triple o de mayor orden (OR 2,60; IC95% 1,57; 4,31), embarazo gemelar (OR 1,14; IC95% 1,00; 1,30), unión estable (OR 1,44; IC95% 1,37; 1,53), escolaridad entre 1 y 7 años (OR 1,26; IC95% 1,14; 1,39), 8 a 11 años (OR 1,20; IC95% 1,11; 1,29), edad materna entre 35 y 49 años (OR 1,19; IC95% 1,11; 1,28), y raza/color de piel amarilla (OR 1,81; IC95% 1,47; 2,24) y negra (OR 1,16; IC95% 1,06; 1,27).

**Conclusión:**

Las características maternas individuales y los factores territoriales se asociaron con la ocurrencia de cardiopatías congénitas, evidenciando perfiles poblacionales y áreas prioritarias para acciones de salud.

**Ethical aspects**

This research used public domain anonymized databases.

## Introduction 

Congenital anomalies, also referred to as malformations or congenital defects, are structural or functional alterations that occur during intrauterine life and may be detected during prenatal care, at birth, or throughout childhood. These conditions constitute a significant public health problem, with considerable impact on child health (1).

Congenital heart diseases account for approximately one-third of all congenital anomalies and stand out as one of the leading causes of infant morbidity and mortality worldwide, with a global prevalence estimated at 9 per 1 thousand live births (2). It is estimated that, in Brazil, more than 25 thousand new cases occur annually, with a higher concentration in the Southeast (3). 

The etiology of these diseases involves genetic, environmental, and socioeconomic factors. Maternal characteristics, such as advanced age, multiple pregnancies, and specific obstetric conditions, are associated with an increased risk of occurrence (4). In addition, territory plays a key role in determining risk and access to health services, especially in urban contexts marked by inequalities (5).

Given their magnitude and impact, congenital heart diseases have been included in the list of congenital anomalies prioritized for monitoring in Brazil by the congenital anomaly surveillance system, reinforcing the need for investigations that support surveillance, prevention, and health actions (6). Understanding territorial patterns and the determinants of these malformations across different local contexts is essential.

The municipality of São Paulo is marked by pronounced social and spatial inequalities, reflected in the distribution of health services, urban infrastructure, and exposure to environmental factors (5,7,8). This scenario may influence both the occurrence and the diagnosis of congenital heart diseases. 

This study proposed integrating spatial analysis at the district level into a population-based cross-sectional study. This approach enabled the identification of territorial risk patterns, the inclusion of the geographic dimension as an explanatory variable in the statistical model, and a deeper understanding of the social and obstetric determinants of congenital heart disease in one of the country’s largest urban centers. 

This study aimed to identify spatial clusters of risk and analyze maternal sociodemographic and obstetric factors associated with the occurrence of congenital heart diseases in the municipality of São Paulo between 2012 and 2022.

## Methods 

### Study design

A population-based cross-sectional study was conducted, including an ecological spatial analysis, based on secondary data. The investigation was carried out in the municipality of São Paulo and encompassed all resident live births between 2012 and 2022.

### Setting 

The municipality of São Paulo is located in the state of São Paulo, in the Southeast of Brazil. With a total area of 1,521 km^2^, it is the most populous city in the country, with 11,451,999 inhabitants in 2022, according to the Brazilian Institute of Geography and Statistics (*Instituto Brasileiro de Geografia e Estatística*, IBGE) (9). The municipality of São Paulo is one of the main economic, political, and cultural centers in Brazil and is characterized by substantial socioeconomic inequalities and variations in the availability of public services (10,11). 

**Figure 1:**
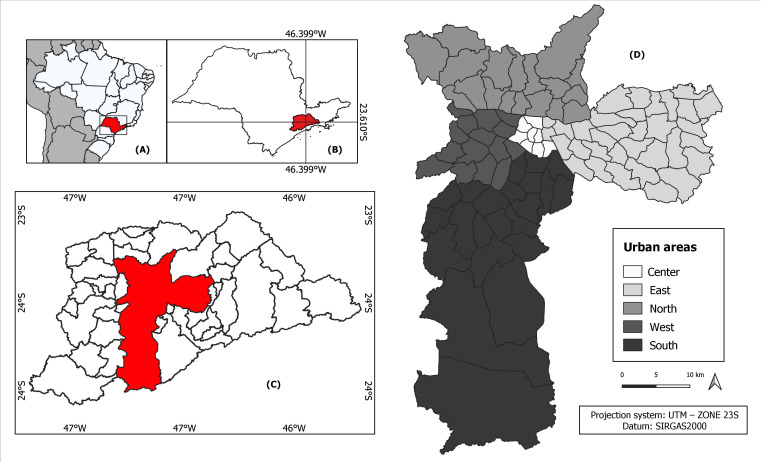
Location of the study area: (A) Brazil; (B) State of São Paulo; (C) São Paulo Metropolitan Region; (D) Municipality of São Paulo

Administratively, the municipality is subdivided into 96 districts, grouped into five urban zones: Center, North, South, East, and West (12) (Figure 1).

### Participants 

All resident live births in the municipality of São Paulo from 2012 to 2022 were included, according to records from the Brazilian Live Birth Information System (*Sistema de Informações sobre Nascidos* Vivos, SINASC), which were made publicly available and anonymized by the Municipal Health Department and extracted on 3 May 2025 (13).

The outcomes were defined as newborns diagnosed with congenital heart disease, based on completion of the specific field on the live birth certificate using codes Q20–Q28 of the International Statistical Classification of Diseases and Related Health Problems 10th Revision. The comparison group consisted of all live births in the same period and location without a record of this condition. 

### Variables 

The outcome variable was the occurrence of congenital heart disease. Independent variables included maternal sociodemographic and obstetric characteristics: age group (≤19, 20–34, 35–49, ≥50 years), education level (none, 1–7, 8–11, ≥12 years of education), race/skin color (White, Brown [Brazilian mixed race], Black, Asian, Indigenous), marital status (single, married, widowed, divorced, stable union), type of pregnancy (single, twin, triple or higher-order), gestational age at birth (preterm, term, postterm), and number of prenatal consultations (none, <7, ≥7). A binary variable was also included representing the mother’s area of residence, classified as within or outside spatial clusters of risk identified in the spatial analysis.

The race/skin color variable was used as recorded in the SINASC, following the official nomenclature also adopted by the IBGE. In the spatial analysis, the term “cluster” referred to the identified risk areas.

### Bias control and study size

To reduce potential sources of bias, all available birth records for the period were used, minimizing selection bias and eliminating the need for prior sample size calculation. Multivariable models were used to adjust for covariates and control for confounding. Including the variable for area of residence indirectly incorporates contextual territorial factors, partially reducing ecological bias.

### Spatial analysis

Spatial analysis was performed using the flexible spatial scan technique with a Poisson model. This model estimated, for each geographic area, the expected number of cases based on the average occurrence rate in the population and the population size of each area, allowing comparison with observed cases and identification of spatial clusters with significantly elevated risk, accommodating irregular geographic shapes and overcoming limitations of traditional models based on circular windows (14). The statistical significance of clusters was assessed using the likelihood ratio test with Monte Carlo simulations (p-value<0.050).

Cases were geocoded using the administrative district codes of the mother’s residence; records without valid geographic correspondence were excluded. Identified risk clusters were classified as primary or secondary based on the ordering of likelihood ratios obtained in the analysis. For each cluster, the ratio between observed and expected cases and the simulation-derived p-value were considered.

Analyses were performed in R software version 4.2.2 (15) using the “rflexscan” package (16), with district centroids as the spatial reference and a queen-contiguity neighborhood matrix. The map was produced in QGIS using the municipal administrative district grid of the municipality of São Paulo, obtained from the São Paulo Municipal Government (17).

Based on the clusters identified, a binary variable “area of residence” was created, indicating whether the mother’s household was located within or outside a spatial cluster of risk. This variable was included as a territorial proxy in the individual models.

### Statistical methods

Statistical analyses were performed in R software, version 4.2.2 (15), using the base glm() function to fit binary logistic regression models. Initially, a descriptive analysis of sociodemographic and obstetric variables was performed, and groups were compared using Pearson’s chi-square test. For the maternal age group, due to low frequencies in some categories, the chi-square test with Monte Carlo simulation (2,000 replications) was applied. A significance level of 5% (p-value<0.050) was adopted for all analyses.

Univariate and multivariate logistic regression models were used. In the univariate analysis, each variable was examined individually with respect to the outcome, and those with a p-value<0.200 were considered for inclusion in the multivariate model, regardless of the width of the confidence interval. Estimates were expressed as *odds ratio* (OR) with corresponding 95% confidence intervals (95%CI). Records with missing data in any of the variables included in the models were excluded from the analyses.

## Results 

Between 2012 and 2022, a total of 1,777,316 live births were recorded among residents of São Paulo. Among these, 9,293 cases of congenital heart diseases were identified, corresponding to a prevalence of 5.23 per one thousand live births.

**Figure 2:**
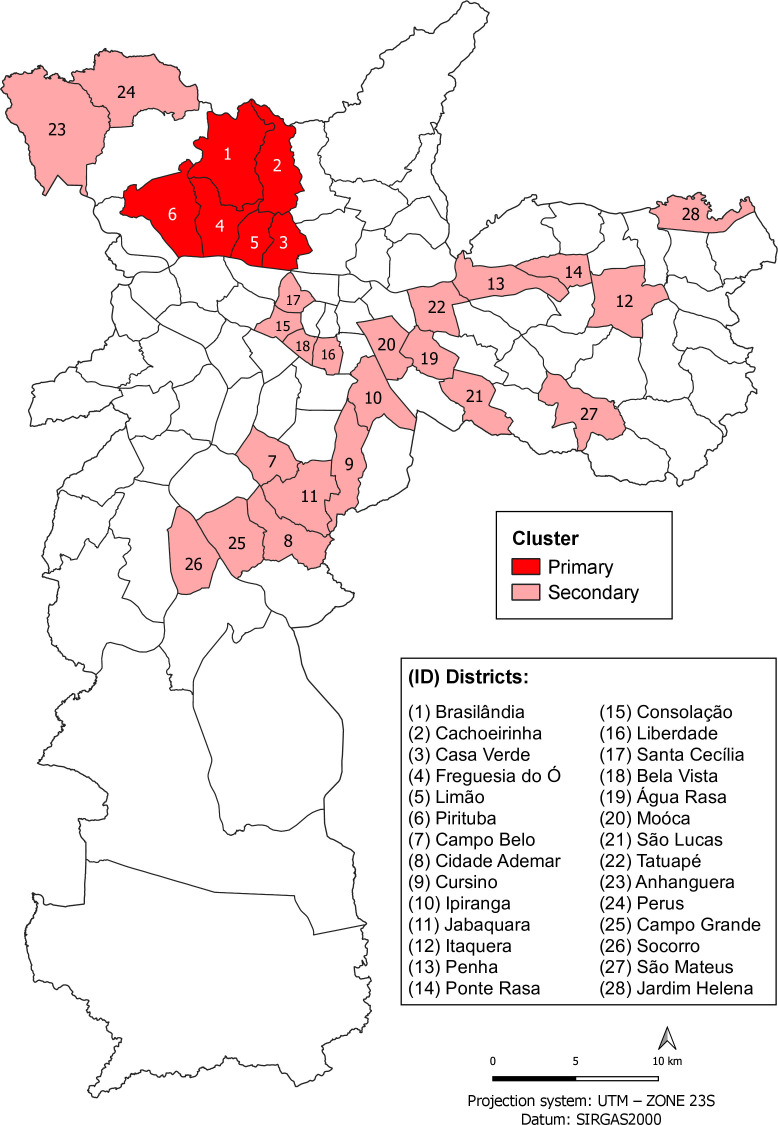
Spatial clusters of congenital heart diseases identified by spatial scan analysis. São Paulo, 2012–2022 (n=9)

Spatial analysis identified clusters of risk across the municipality’s regions (Figure 2). The primary cluster, located in the Northern area, observed 1,696 cases, compared with 676 expected (relative risk 2.51; p-value<0.001). Eight secondary clusters were detected, encompassing 23 districts, with relative risks ranging from 1.36 to 1.75 (p-value<0.050) (Table 1).

**Table 1:** Relative risk (RR) of spatial clusters of congenital heart diseases, based on observed and expected cases. São Paulo, 2012–2022 (n=9,233)

Identifier/districts	Cluster	Observed cases	Expected cases	RR	p-value
(1) Brasilândia, (2) Cachoeirinha, (3) Casa Verde, (4) Freguesia do Ó, (5) Limão, (6) Pirituba	Primary	1,696	676	2.51	0.001
(7) Campo Belo, (8) Cidade Ademar, (9) Cursino, (10) Ipiranga, (11) Jabaquara	Secondary	876	500	1.75	0.001
(12) Itaquera, (13) Penha, (14) Ponte Rasa	Secondary	685	420	1.63	0.001
(15) Consolação, (16) Liberdade, (17) Santa Cecília, (18) Bela Vista	Secondary	430	303	1.42	0.001
(19) Água Rasa, (20) Moóca, (21) São Lucas, (22) Tatuapé	Secondary	259	178	1.45	0.001
(23) Anhanguera, (24) Perus	Secondary	166	116	1.43	0.016
(25) Campo Grande, (26) Socorro	Secondary	189	139	1.36	0.045
(27) São Mateus	Secondary	102	63	1.63	0.009
(28) Jardim Helena	Secondary	81	47	1.74	0.007

The study population was described according to the presence or absence of congenital heart diseases at birth, based on sociodemographic and obstetric characteristics (Table 2). In both groups, the predominant maternal age category was 20–34 years (64.4% in cases vs. 68.6% in the comparison group). Eight to 11 years of schooling was the most frequent category in both groups (60.3% among cases and 61.5% in the comparison group). The largest proportion of mothers was White (48.6%), whereas Indigenous mothers represented the smallest proportion (0.4%). Single mothers accounted for 40.5% of cases and 43.2% of the comparison group, followed by married mothers (34.8% and 37.0%, respectively).

**Table 2:** Sociodemographic and obstetric characteristics, according to the presence or absence of congenital heart disease. São Paulo, 2012–2022 (n=1,757,073)

Variable	With congenital heart disease	Without congenital heart disease	p-value
	n (%)	n (%)	
**Age group (years)**			<0,001
≤19	1.038 (11,2)	198.067 (11,3)	
20-34	5.975 (64,6)	1.205.645 (68,6)	
35-49	2.232 (24,1)	353.107 (20,1)	
≥50	2 (0,0)	254 (0,0)	
**Education level (years of schooling)**			<0,013
None	10 (0,1)	1.750 (0,1)	
1-7	904 (9,8)	156.278 (8,9)	
8-11	5.574 (60,3)	1.080.870 (61,5)	
≥12	2.759 (29,8)	518.175 (29,5)	
**Maternal race/skin color**			<0,001
White	4.494 (48,6)	857.583 (48,8)	
Black	884 (9,6)	140.894 (8,0)	
Asian	142 (1,5)	20.770 (1,2)	
Brown	3.686 (39,9)	731.615 (41,6)	
Indigenous	41 (0,4)	6.211 (0,4)	
**Marital status**			<0,001
Single	3.745 (40,5)	758.540 (43,2)	
Married	3.219 (34,8)	649.486 (37,0)	
Widowed	15 (0,2)	2.478 (0,1)	
Divorced	152 (1,6)	27.167 (1,5)	
Stable union	2.116 (22,9)	319.402 (18,2)	
**Pregnancy**			<0,001
Preterm	2.256 (24,4)	188.237 (10,7)	
Full-term birth	6.908 (74,7)	1.550.640 (88,3)	
Postterm	83 (0,9)	18.196 (1,0)	
**Number of prenatal consultations**			<0,001
None	106 (1,1)	18.165 (1,0)	
<7	2.118 (22,9)	349.744 (19,9)	
≥7	7.023 (75,9)	1.389.164 (79,1)	
**Type of pregnancy**			<0,001
Singleton	8.727 (94,4)	1.710.039 (97,3)	
Twin	473 (5,1)	45.786 (2,6)	
Triplet or higher-order	47 (0,5)	1.248 (0,1)	
**Area of residence**			<0,001
Outside the cluster	4.970 (53,7)	1.323.803 (75,3)	
Inside the cluster	4.277 (46,3)	433.270 (24,7)	

The analysis of obstetric factors showed that preterm birth was more frequent among newborns with congenital heart disease (24.4% vs. 10.7%). Although most births occurred at term, this proportion was lower in the outcome group (74.7% vs. 88.3%). Seven or more prenatal care visits predominated in both groups (75.9% and 79.1%), whereas fewer than seven visits were observed in 22.9% of cases and 19.9% of the comparison group. Singleton pregnancies were the most frequent (94.4% in cases and 97.3% in the comparison group), although twin pregnancies (5.1% vs. 2.6%) and triple or higher-order pregnancies (0.5% vs. 0.1%) were more common among newborns with congenital heart disease. Residence in spatial clusters of risk was also more frequent in this group (46.3% vs. 24.7%).

Results from the univariate and multivariate logistic regression analyses, including estimates for cases and the comparison group, are presented in Table 3. Mothers aged 35–49 years had higher odds of having an infant with congenital heart disease compared with those aged 20–34 years (adjusted OR 1.19; 95%CI 1.11; 1.28). In the initial model, mothers with 1–7 years of schooling had higher odds than those with 12 or more years of schooling (unadjusted OR 1.09; 95%CI 1.01; 1.17), with no significant association in the other categories. After adjustment, the odds remained elevated for 1–7 years of schooling (adjusted OR 1.26; 95%CI 1.14; 1.39), and the association became significant for 8–11 years of schooling as well (adjusted OR 1.20; 95%CI 1.11; 1.29).

**Table 3:** Unadjusted and adjusted odds ratio (OR) with 95% confidence intervals (95%CI) for congenital heart diseases, according to sociodemographic and obstetric variables. São Paulo, 2012–2022 (n=1,757,073)

Variable	Unadjusted OR (95%CI)	p-value	Adjusted OR (95%CI)
**Age group (years)**			
≤19	1,06 (0,99; 1,13)	0,097	1,00 (0,94; 1,08)
20-34 (reference)	-	-	-
35-49	1,28 (1,21; 1,34)	0,000	1,19 (1,11; 1,28)
≥50	1,59 (0,26; 4,95)	0,514	-
**Education level (years of schooling**			
None	1,07 (0,54; 1,89)	0,824	-
1-7	1,09 (1,01; 1,17)	0,031	1,26 (1,14; 1,39)
8-11	0,97 (0,93; 1,01)	0,171	1,20 (1,11; 1,29)
≥12 (reference)	-	-	-
**Maternal race/skin color**			
White (referência)	-	-	-
Black	1,20 (1,11; 1,29)	0,000	1,16 (1,06; 1,27)
Asian	1,30 (1,10; 1,54)	0,002	1,81 (1,47; 2,24)
Brown	0,96 (0,92; 1,00)	0,077	1,03 (0,98; 1,09)
Indigenous	1,26 (0,91; 1,69)	0,142	1,07 (0,77; 1,48)
**Marital status**			
Single(reference)	-	-	-
Married	1,00 (0,96; 1,05)	0,873	-
Widowed	1,23 (0,70; 1,96)	0,432	-
Divorced	1,13 (0,96; 1,33)	0,132	1,11 (0,94; 1,32)
Stable union	1,34 (1,27; 1,42)	0,000	1,44 (1,37; 1,53)
**Pregnancy**			
Preterm	2,69 (2,56; 2,82)	0,000	2,61 (2,45; 2,78)
Term birth (reference)	-	-	-
Postterm	1,02 (0,82; 1,26)	0,831	-
**Number of prenatal consultations**			
None	1,15 (0,95; 1,39)	0,144	0,87 (0,71; 1,07)
<7	1,20 (1,14; 1,26)	0,000	1,03 (0,97; 1,09)
≥7 (reference)	-	-	-
Type of pregnancy			
Single (reference	-	-	-
Twin	2,02 (1,84; 2,22)	0,000	1,14 (1,00; 1,30)
Triplet or higher-order	7,38 (5,44; 9,76)	0,000	2,60 (1,57; 4,31)
**Area of residence**			
Outside the cluster (reference)	-	-	-
Inside the cluster	2,63 (2,52; 2,74)	0,000	2,97 (2,82; 3,12)

Maternal race/skin color remained significantly associated with the outcome. Compared with White mothers, those identifying as Asian (adjusted OR 1.81; 95%CI 1.47; 2.24) and Black (adjusted OR 1.16; 95%CI 1.06; 1.27) had higher odds of having an infant with congenital heart disease. Mothers in a stable union had higher odds of the outcome (adjusted OR 1.44; 95%CI 1.37; 1.53), whereas for divorced mothers the association was no longer significant after adjustment.

Preterm pregnancies had 2.6-fold higher odds of congenital heart disease compared with the reference group (adjusted OR 2.61; 95%CI 2.45; 2.78). In the initial model, fewer than seven prenatal consultations were associated with increased odds of congenital heart disease (unadjusted OR 1.20; 95%CI 1.14; 1.26), but this association did not remain after adjustment. Twin pregnancies showed higher odds compared with singleton pregnancies (adjusted OR 1.14; 95%CI 1.00; 1.30). The odds were more than doubled in triple or higher-order pregnancies (adjusted OR 2.60; 95%CI 1.57; 4.31). Mothers living in areas previously identified as spatial clusters of risk had nearly three times the odds of having an infant with congenital heart disease compared with those living outside these areas (adjusted OR 2.97; 95%CI 2.82; 3.12).

## Discussion 

The findings of this study indicate that maternal, obstetric, and spatial characteristics are associated with the occurrence of congenital heart disease in the municipality of São Paulo. Higher odds were observed among mothers of advanced maternal age, with low education levels, identifying as Asian or Black, in a stable union, with preterm and multiple pregnancies, and residing within spatial clusters of risk.

The spatial analysis revealed a pattern of inequality in the distribution of congenital heart disease risk across the municipality. A prominent high-risk cluster was located in an area characterized by a high proportion of households in favelas and poor urban communities and a concentration of low-income populations (5). This finding reinforces evidence that social and environmental vulnerability is associated with poorer health outcomes, including the occurrence of congenital anomalies (7,18). 

Maternal and obstetric characteristics proved relevant for understanding patterns of occurrence of these malformations. Advanced maternal age was one of the associated factors, consistent with previous studies linking later pregnancies to increased chromosomal abnormalities, such as Down syndrome (19). The association between lower education level and higher odds—although of moderate impact—underscores the role of schooling as a marker of socioeconomic context, reflecting inequalities in access to information, the quality of prenatal care, and health services (20,21). 

Analysis of maternal race/skin color showed that Asian and Black mothers had higher odds of congenital heart disease compared with White mothers, though the impact was lower among Black mothers. Studies in different contexts have reported similar results. In Asian populations, a higher prevalence of these anomalies has been observed, possibly associated with specific genetic and environmental factors (2). In the Brazilian context, studies suggest that Black mothers are more exposed to social vulnerabilities, such as low education level and fewer prenatal care consultations—conditions frequently associated with adverse perinatal outcomes (22). 

Maternal marital status was also associated with the occurrence of congenital heart disease, with higher odds among mothers in a stable union compared with single mothers. However, there was insufficient evidence to support a direct causal relationship, and it is possible that this variable acts as an indirect marker of contextual characteristics, such as demographic profiles or patterns of access to health services. This finding should be interpreted with caution and further explored in studies that incorporate additional information on social and family context.

Among the obstetric factors analyzed, prematurity had one of the strongest associations. This finding corroborates research indicating preterm birth as an important risk factor for cardiac malformations (23). This effect may reflect disruptions in fetal development during critical periods of gestation, increasing vulnerability to structural defects (23).

In this study, an insufficient number of prenatal care consultations was not associated with the outcome after model adjustment. Nonetheless, evidence indicates that inadequate antenatal care is associated with a higher risk of congenital anomalies and other adverse neonatal outcomes (24). In Brazil, the Ministry of Health recommends at least six prenatal consultations, which are considered essential for reducing maternal and infant morbidity and mortality and for the early detection of complications (25).

Multiple pregnancies, particularly higher-order multiple pregnancies, were also associated with increased odds of congenital heart diseases, a finding consistent with the literature, which has reported greater vulnerability and greater complexity in the clinical management of these cases (26). This result underscores the importance of intensified prenatal strategies and specialized follow-up for multiple pregnancies, aimed at prevention and early detection of these malformations.

Maternal area of residence proved to be an important factor associated with the occurrence of congenital heart diseases, with higher odds among mothers living in areas identified as spatial clusters of risk. This finding reinforces that territory is not merely the setting in which events occur, but also an active determinant of outcomes.

Using the variable “residence in a spatial risk-cluster area” as a territorial proxy enabled integration of the spatial dimension into the individual-level analysis. Although this study did not include specific indicators of the social or physical environment of residential areas, evidence shows that exposures such as air pollution, chemical agents, and barriers to access to health services are associated with adverse health outcomes, including congenital anomalies (7,8). The association observed between area of residence and congenital heart diseases may reflect the cumulative effect of contextual exposures.

Although widely applied in various areas of public health, spatial analysis remains underexplored in the study of congenital heart diseases. This approach has shown promise in identifying geographic risk patterns and in generating hypotheses about spatial determinants of the condition (27,28). In large urban centers such as the municipality of São Paulo, its use may represent a strategic tool to support surveillance and health action planning.

Among the limitations of this study, the inability to geocode by residential address was notable, limiting spatial analysis to the administrative district level. Although this unit captures relevant spatial patterns, its scale may mask important within-district heterogeneity, particularly in large and densely populated areas. 

Another limitation relates to the data source used. Although the SINASC is widely used in epidemiological research, it is a secondary database that may underreport mild cases, limit the collection of contextual information, and fail to capture diagnoses established later. Such gaps may introduce imprecision into analyses and hinder the inclusion of explanatory variables related to the environmental context, thereby reducing the analysis’s sensitivity. 

The use of an aggregated territorial variable (residence in a spatial risk-cluster area) as a proxy for spatial context does not substitute for the direct use of environmental or socioeconomic indicators. The absence of these indicators in the analyses limited the ability to control for potential confounding factors related to territory, representing a methodological limitation of the study. Future studies may deepen this approach by integrating more detailed environmental data and incorporating the spatial component directly into the analytical models. This will allow the identification of risk patterns not explained solely by individual characteristics, thereby enhancing understanding of the determinants of congenital heart disease.

Finally, a limitation inherent to the study design should be highlighted. As this was a cross-sectional study, the findings do not allow causal inference, and caution is necessary when discussing potential implications for public policies or health interventions.

The results of this study indicated that maternal sociodemographic characteristics, obstetric factors, and the area of residence are associated with the occurrence of congenital heart diseases in the municipality of São Paulo. By highlighting vulnerable maternal profiles and the unequal territorial distribution of risk, the study provided evidence to support congenital anomaly surveillance and the planning of priority public health actions within the Brazilian Unified Health System (*Sistema Único de Saúde*, SUS) to prevent, detect early, and care for congenital heart diseases. Such evidence is particularly relevant for reducing morbidity and mortality associated with these conditions and for improving maternal and child health interventions.
