# Supplementary material for: Congenital heart diseases: population-based cross-sectional study and spatial analysis, São Paulo, 2012–2022
Source: Epidemiol Serv Saude. 2026 Mar 16;35:e20250519. doi: 10.1590/S2237-96222026v35e20250519.en (PMC12991397; doi:10.1590/S2237-96222026v35e20250519.en)
Supplement: Translation

# Translation: Cardiopatias congênitas: estudo transversal de base populacional e análise espacial, município de São Paulo, 2012-2022

**DOI:** 10.1590/S2237-96222026v35e20250519.pt

**Authors:** Amarílis Bahia Bezerra, Ligia Vizeu Barrozo, Alfredo Pereira de Queiroz

**Affiliations:** 1 Universidade de São Paulo, Departamento de Geografia, São Paulo, SP, Brasil

**Keywords:** Cardiopatias Congênitas, Análise Espacial, Saúde Materno-Infantil, Epidemiologia, Estudos Transversais

## Abstract

**Objetivos:**

Identificar agrupamentos espaciais de risco e analisar os fatores sociodemográficos e obstétricos maternos associados à ocorrência de cardiopatias congênitas no município de São Paulo entre 2012 e 2022.

**Método:**

Estudo transversal de base populacional e análise espacial de caráter ecológico, utilizando dados do Sistema de Informações sobre Nascidos Vivos, de 2012 a 2022, no município de São Paulo. Os desfechos foram recém-nascidos com diagnóstico de cardiopatia congênita (10ª revisão da Classificação Internacional de Doenças: Q20 a Q28) e o grupo de comparação, os demais nascidos vivos. Aplicaram-se varredura espacial e regressão logística para estimar razões de chances (*odds ratio*, OR) e intervalos de confiança de 95% (IC95%).

**Resultados:**

Foram registrados 1.777.316 nascidos vivos, dos quais 9.293 apresentaram cardiopatias congênitas. A análise espacial identificou um agrupamento de casos na Zona Norte do município. A ocorrência esteve associada a: residência em áreas de agrupamento espacial (OR 2,97; IC95% 2,82; 3,12), gestações pré-termo (OR 2,61; IC95% 2,45; 2,78), gravidez tripla ou mais (OR 2,60; IC95% 1,57; 4,31), gravidez dupla (OR 1,14; IC95% 1,00; 1,30), união estável (OR 1,44; IC95% 1,37; 1,53), escolaridade entre 1 e 7 anos (OR 1,26; IC95% 1,14; 1,39), 8 a 11 anos (OR 1,20; IC95% 1,11; 1,29), idade entre 35 e 49 anos (OR 1,19; IC95% 1,11; 1,28), raça/cor da pele amarela (OR 1,81; IC95% 1,47; 2,24) e preta (OR 1,16; IC95% 1,06; 1,27).

**Conclusão:**

Características maternas individuais e fatores territoriais estiveram associados à ocorrência de cardiopatias congênitas, evidenciando perfis populacionais e áreas prioritárias para ações em saúde.

**Aspectos éticos**

Esta pesquisa utilizou bancos de dados de domínio público e anonimizados.

## Introdução 

As anomalias congênitas, também chamadas de malformações ou defeitos congênitos, referem-se a alterações estruturais ou funcionais que ocorrem durante a vida intrauterina e podem ser detectadas no pré-natal, no nascimento ou ao longo da infância. Essas condições configuram relevante problema de saúde pública, com impacto considerável na saúde infantil (1).

As cardiopatias congênitas representam aproximadamente um terço de todas as anomalias congênitas e destacam-se entre as principais causas de morbimortalidade infantil no mundo, com prevalência global estimada de 9 por 1 mil nascidos vivos (2). Estima-se que, no Brasil, ocorram mais de 25 mil novos casos anuais, com maior concentração na região Sudeste (3). 

A etiologia dessas doenças envolve fatores genéticos, ambientais e socioeconômicos. Características maternas, como idade avançada, gestações múltiplas e condições obstétricas específicas, estão associadas ao maior risco de ocorrência (4). Além disso, o território exerce papel fundamental na determinação do risco e do acesso aos serviços de saúde, especialmente em contextos urbanos marcados por desigualdades (5).

Diante de sua magnitude e impacto, as cardiopatias congênitas foram incluídas na lista de defeitos congênitos considerados prioritários para monitoramento, no Brasil, pela vigilância de anomalias congênitas, reforçando a necessidade de investigações que subsidiem ações de vigilância, prevenção e cuidado (6). Compreender os padrões territoriais e os determinantes associados à ocorrência dessas malformações em diferentes realidades locais torna-se fundamental.

O município de São Paulo é marcado por expressivas desigualdades sociais e espaciais, refletidas na distribuição de serviços de saúde, infraestrutura urbana e exposição a fatores ambientais (5,7,8). Esse cenário pode influenciar tanto a ocorrência quanto o diagnóstico das cardiopatias congênitas. 

Este estudo propôs a integração da análise espacial em nível distrital a um estudo transversal de base populacional. Essa abordagem permitiu identificar padrões territoriais de risco, incorporar a dimensão geográfica como variável explicativa no modelo estatístico e aprofundar a compreensão sobre os determinantes sociais e obstétricos associados à ocorrência de cardiopatias congênitas em um dos maiores centros urbanos do país. 

Este estudo teve como objetivos identificar agrupamentos espaciais de risco e analisar os fatores sociodemográficos e obstétricos maternos associados à ocorrência de cardiopatias congênitas no município de São Paulo entre 2012 e 2022.

## Métodos 

### Delineamento 

Trata-se de estudo transversal de base populacional, incluindo uma análise espacial de caráter ecológico, baseados em dados secundários. A investigação foi conduzida no município de São Paulo e abrangeu todos os nascidos vivos residentes entre 2012 e 2022.

### Contexto 

O município de São Paulo está localizado no estado de São Paulo, na região Sudeste do Brasil. Com extensão territorial de 1.521 km^2^, é a cidade mais populosa do país, com 11.451.999 habitantes em 2022, segundo o Instituto Brasileiro de Geografia e Estatística (9). O município de São Paulo é um dos principais centros econômicos, políticos e culturais do Brasil, concentrando grandes desigualdades socioeconômicas e variações na oferta de serviços públicos (10,11). 

O município está administrativamente subdividido em 96 distritos, agrupados em cinco zonas urbanas: Central, Norte, Sul, Leste e Oeste (12) (Figura 1).

**Figura 1:** Localização da área de estudo: (A) Brasil; (B) estado de São Paulo; (C) região metropolitana de São Paulo; (D) município de São Paulo

### Participantes 

Foram incluídos todos os nascidos vivos residentes no município de São Paulo no período 2012-2022, conforme os registros do Sistema de Informações sobre Nascidos Vivos, disponibilizados pela Secretaria Municipal da Saúde em formato público e anonimizado, extraídos em 3 de maio de 2025 (13).

Os desfechos foram definidos como recém-nascidos com diagnóstico de cardiopatia congênita, conforme o preenchimento do campo específico da declaração de nascido vivo com base nos códigos Q20 a Q28 da 10ª revisão da Classificação Internacional de Doenças. O grupo de comparação foi composto por todos os nascidos vivos no mesmo período e local, sem registro dessa condição. 

### Variáveis 

A variável desfecho foi a ocorrência de cardiopatia congênita. As variáveis independentes incluíram características sociodemográficas e obstétricas da mãe: faixa etária (≤19, 20-34, 35-49, ≥50 anos), escolaridade (nenhuma, 1-7, 8-11, ≥12 anos de estudo), raça/cor da pele (branca, parda, preta, amarela, indígena), estado civil (solteira, casada, viúva, divorciada, união estável), tipo de gestação (única, dupla, tripla ou mais), idade gestacional ao parto (pré-termo, a termo, pós-termo), número de consultas de pré-natal (nenhuma, <7, ≥7). Também foi incluída uma variável binária representando a área de residência da mãe, classificada como dentro ou fora dos agrupamentos espaciais de risco, identificados na análise espacial.

A variável raça/cor da pele foi utilizada conforme o registro padronizado do Sistema de Informações sobre Nascidos Vivos, em consonância com a nomenclatura oficial também adotada pelo Instituto Brasileiro de Geografia e Estatística. Para a análise espacial, adotou-se o termo “agrupamento”, em referência às áreas de risco identificadas.

### Controle de viés e tamanho do estudo

Para reduzir potenciais fontes de viés, foi utilizada a totalidade dos registros de nascimentos disponíveis no período, minimizando viés de seleção e dispensando cálculo amostral prévio. Modelos multivariados foram empregados para ajustar os efeitos das covariáveis e controlar confusão estatística. A inclusão da variável de área de residência permitiu incorporar, de forma indireta, fatores contextuais relacionados ao território, o que reduziu parcialmente o viés ecológico.

### Análise espacial

A análise espacial foi realizada com a técnica de varredura espacial flexível, com o modelo de Poisson. Esse modelo estimou, para cada área geográfica, o número de casos esperados a partir da taxa média de ocorrência na população e do tamanho populacional de cada área, permitindo comparar esses valores com os casos observados e identificar aglomerados espaciais com risco significativamente elevado, abrangendo formatos geográficos irregulares e superando a limitação dos modelos tradicionais baseados em janelas circulares (14). A significância estatística dos agrupamentos foi avaliada por meio do teste da razão de verossimilhança, com simulações de Monte Carlo (p-valor<0,050).

Os casos foram geocodificados a partir dos códigos dos distritos administrativos de residência da mãe, sendo excluídos os registros sem correspondência geográfica válida. Os agrupamentos de risco identificados foram classificados como primários e secundários, conforme ordenação da razão de verossimilhança obtida na análise. Para cada agrupamento, foram consideradas a razão entre casos observados e esperados e o p-valor estimado por simulação.

As análises foram realizadas no software R versão 4.2.2 (15), com uso do pacote “rflexscan” (16), utilizando os centroides dos distritos como referência espacial e matriz de vizinhança do tipo queen. O mapa foi elaborado com o auxílio do software QGIS, com base na malha territorial dos distritos administrativos do município de São Paulo, obtida junto à Prefeitura de São Paulo (17).

A partir dos agrupamentos identificados, foi criada a variável binária “área de residência”, a qual indicava se o domicílio da mãe estava localizado dentro ou fora de um agrupamento espacial de risco. Essa variável foi incluída como proxy territorial nos modelos individuais.

### Análise estatística

A análise estatística foi conduzida no software R, versão 4.2.2 (15), utilizando a função glm() do pacote base para ajuste da regressão logística binária. Inicialmente, foi realizada uma análise descritiva das variáveis sociodemográficas e obstétricas, com comparação entre os grupos, por meio do teste do qui-quadrado de Pearson. Para a variável faixa etária materna, devido à baixa frequência em algumas categorias, utilizou-se o teste do qui-quadrado com simulação de Monte Carlo (2 mil repetições). Adotou-se o nível de significância de 5% (p-valor<0,050) para todas as análises.

Foram aplicados modelos de regressão logística univariada e multivariada. No modelo univariado, cada variável foi analisada isoladamente em relação ao desfecho, e aquelas com p-valor<0,200 foram consideradas para inclusão no modelo múltiplo, independentemente da amplitude do intervalo de confiança. As estimativas foram expressas como razões de chance (*odds ratio*, OR), com respectivos intervalos de confiança de 95% (IC95%). Observações com informações ausentes em quaisquer das variáveis incluídas nos modelos foram excluídas das análises.

## Resultados 

Entre 2012 e 2022, foram registrados 1.777.316 nascidos vivos residentes no município de São Paulo. Entre esses, foram identificados 9.293 casos de cardiopatias congênitas, o que correspondeu à prevalência de 5,23 por 1 mil nascidos vivos.

A análise espacial identificou agrupamentos de risco em diferentes regiões do município (Figura 2). O agrupamento primário, localizado na Zona Norte, registrou 1.696 casos versus 676 esperados (risco relativo 2,51; p-valor<0,001). Foram detectados 8 agrupamentos secundários, distribuídos em 23 distritos, com riscos relativos variando de 1,36-1,75 (p-valor<0,050) (Tabela 1).

**Tabela 1:** Risco relativo (RR) dos agrupamentos espaciais de cardiopatias congênitas, segundo casos observados e esperados. Município de São Paulo, 2012-2022 (n=9.233)

Identificador/distritos	Agrupamento	Casos observados	Casos esperados	RR	p-valor
(1) Brasilândia, (2) Cachoeirinha, (3) Casa Verde, (4) Freguesia do Ó, (5) Limão, (6) Pirituba	Primário	1.696	676	2,51	0,001
(7) Campo Belo, (8) Cidade Ademar, (9) Cursino, (10) Ipiranga, (11) Jabaquara	Secundário	876	500	1,75	0,001
(12) Itaquera, (13) Penha, (14) Ponte Rasa	Secundário	685	420	1,63	0,001
(15) Consolação, (16) Liberdade, (17) Santa Cecília, (18) Bela Vista	Secundário	430	303	1,42	0,001
(19) Água Rasa, (20) Moóca, (21) São Lucas, (22) Tatuapé	Secundário	259	178	1,45	0,001
(23) Anhanguera, (24) Perus	Secundário	166	116	1,43	0,016
(25) Campo Grande, (26) Socorro	Secundário	189	139	1,36	0,045
(27) São Mateus	Secundário	102	63	1,63	0,009
(28) Jardim Helena	Secundário	81	47	1,74	0,007

**Figura 2:** Agrupamentos espaciais de cardiopatias congênitas identificados por varredura espacial. Município de São Paulo, 2012-2022 (n=9)

Apresentou-se a descrição da população estudada segundo a presença ou ausência de cardiopatia congênita no nascimento, considerando características sociodemográficas e obstétricas (Tabela 2). Em ambos os grupos, a faixa etária materna predominante foi 20-34 anos (64,4% nos desfechos vs. 68,6% no grupo de comparação). Oito a 11 anos de estudo foi o mais frequente tanto nos desfechos (60,3%) quanto no grupo de comparação (61,5%). A maior proporção foi registrada entre brancas (48,6%), enquanto a frequência de indígenas apresentou a menor proporção (0,4%). Mães solteiras representaram 40,5% nos desfechos e 43,2% no grupo de comparação, seguidas por mães casadas (34,8% e 37,0%).

**Tabela 2:** Características sociodemográficas e obstétricas, segundo presença ou ausência de cardiopatias congênitas. Município de São Paulo, 2012-2022 (n=1.757.073)

Variável	Com cardiopatia congênita	Sem cardiopatia congênita	p-valor
	n (%)	n (%)	
**Faixa etária** (anos)			<0,001
≤19	1.038 (11,2)	198.067 (11,3)	
20-34	5.975 (64,6)	1.205.645 (68,6)	
35-49	2.232 (24,1)	353.107 (20,1)	
≥50	2 (0,0)	254 (0,0)	
**Escolaridade** (**anos de estudos**)			<0,013
Nenhuma	10 (0,1)	1.750 (0,1)	
1-7	904 (9,8)	156.278 (8,9)	
8-11	5.574 (60,3)	1.080.870 (61,5)	
≥12	2.759 (29,8)	518.175 (29,5)	
**Raça/cor da pele da mãe**			<0,001
Branca	4.494 (48,6)	857.583 (48,8)	
Preta	884 (9,6)	140.894 (8,0)	
Amarela	142 (1,5)	20.770 (1,2)	
Parda	3.686 (39,9)	731.615 (41,6)	
Indígena	41 (0,4)	6.211 (0,4)	
**Estado civil**			<0,001
Solteira	3.745 (40,5)	758.540 (43,2)	
Casada	3.219 (34,8)	649.486 (37,0)	
Viúva	15 (0,2)	2.478 (0,1)	
Divorciada	152 (1,6)	27.167 (1,5)	
União estável	2.116 (22,9)	319.402 (18,2)	
Gestação			<0,001
Pré-termo	2.256 (24,4)	188.237 (10,7)	
A termo	6.908 (74,7)	1.550.640 (88,3)	
Pós-termo	83 (0,9)	18.196 (1,0)	
**Número de consultas pré**-natal			<0,001
Nenhuma	106 (1,1)	18.165 (1,0)	
<7	2.118 (22,9)	349.744 (19,9)	
≥7	7.023 (75,9)	1.389.164 (79,1)	
**Tipo de gravidez**			<0,001
Única	8.727 (94,4)	1.710.039 (97,3)	
Dupla	473 (5,1)	45.786 (2,6)	
Tripla ou mais	47 (0,5)	1.248 (0,1)	
**Área de residência**			<0,001
Fora do agrupamento	4.970 (53,7)	1.323.803 (75,3)	
Dentro do agrupamento	4.277 (46,3)	433.270 (24,7)	

A análise dos fatores obstétricos evidenciou que a gestação pré-termo foi mais frequente entre os nascidos com cardiopatia congênita (24,4% vs. 10,7%). Embora a maioria dos nascimentos tenham ocorrido a termo, essa proporção foi menor no grupo de desfechos (74,7% vs. 88,3%). Predominou a realização de 7 ou mais consultas de pré-natal em ambos os grupos (75,9% e 79,1%), enquanto menos de 7 consultas foi observado em 22,9% dos desfechos e 19,9% no grupo de comparação. A gestação única foi predominante (94,4% nos desfechos e 97,3% no grupo de comparação), porém as gestações duplas (5,1% vs. 2,6%) e triplas ou mais (0,5% vs. 0,1%) apresentaram maior proporção no grupo com cardiopatia congênita. A residência em agrupamentos de risco também apresentou maior proporção nesse grupo (46,3% vs. 24,7%).

Apresentaram-se os resultados das análises de regressão logística binária e múltipla, com as estimativas obtidas para os desfechos e o grupo de comparação (Tabela 3). Mães com idade entre 35-49 anos apresentaram maiores chances de ter um filho com cardiopatia congênita em comparação às de 20-34 anos (OR ajustada 1,19; IC95% 1,11; 1,28). No modelo inicial, as mães com 1-7 anos de estudo apresentaram maiores chances em relação às com 12 anos ou mais (OR bruta 1,09; IC95% 1,01; 1,17), não sendo observada associação significativa nas demais categorias. Após ajuste, as chances permaneceram elevadas para 1-7 anos de estudo (OR ajustada 1,26; IC95% 1,14; 1,39) e tornou-se significativo também para 8-11 anos (OR ajustada 1,20; IC95% 1,11; 1,29).

**Tabela 3:** Razões de chance (*odds ratio*, OR) brutas e ajustadas e intervalos de confiança de 95% (IC95%) de cardiopatia congênita, segundo variáveis sociodemográficas e obstétricas. Município de São Paulo, 2012-2022 (n=1.757.073)

Variável	OR bruta (IC95%)	p-valor	OR ajustada (IC95%)
**Faixa etária** (anos)			
≤19	1,06 (0,99; 1,13)	0,097	1,00 (0,94; 1,08)
20-34 (referência)	-	-	-
35-49	1,28 (1,21; 1,34)	0,000	1,19 (1,11; 1,28)
≥50	1,59 (0,26; 4,95)	0,514	-
**Escolaridade** (**anos de estudos**)			
Nenhuma	1,07 (0,54; 1,89)	0,824	-
1-7	1,09 (1,01; 1,17)	0,031	1,26 (1,14; 1,39)
8-11	0,97 (0,93; 1,01)	0,171	1,20 (1,11; 1,29)
≥12 (referência)	-	-	-
**Raça/cor da pele da mãe**			
Branca (referência)	-	-	-
Preta	1,20 (1,11; 1,29)	0,000	1,16 (1,06; 1,27)
Amarela	1,30 (1,10; 1,54)	0,002	1,81 (1,47; 2,24)
Parda	0,96 (0,92; 1,00)	0,077	1,03 (0,98; 1,09)
Indígena	1,26 (0,91; 1,69)	0,142	1,07 (0,77; 1,48)
**Estado civil**			
Solteira (referência)	-	-	-
Casada	1,00 (0,96; 1,05)	0,873	-
Viúva	1,23 (0,70; 1,96)	0,432	-
Divorciada	1,13 (0,96; 1,33)	0,132	1,11 (0,94; 1,32)
União estável	1,34 (1,27; 1,42)	0,000	1,44 (1,37; 1,53)
Gestação			
Pré-termo	2,69 (2,56; 2,82)	0,000	2,61 (2,45; 2,78)
A termo (referência)	-	-	-
Pós-termo	1,02 (0,82; 1,26)	0,831	-
**Número de consultas pré**-natal			
Nenhuma	1,15 (0,95; 1,39)	0,144	0,87 (0,71; 1,07)
<7	1,20 (1,14; 1,26)	0,000	1,03 (0,97; 1,09)
≥7 (referência)	-	-	-
Tipo de gravidez			
Única (referência)	-	-	-
Dupla	2,02 (1,84; 2,22)	0,000	1,14 (1,00; 1,30)
Tripla ou mais	7,38 (5,44; 9,76)	0,000	2,60 (1,57; 4,31)
**Área de residência**			
Fora do agrupamento (referência)	-	-	-
Dentro do agrupamento	2,63 (2,52; 2,74)	0,000	2,97 (2,82; 3,12)

A variável raça/cor da pele manteve associação significativa. Em relação às mães brancas, as de raça/cor da pele amarela (OR ajustada 1,81; IC95% 1,47; 2,24) e preta (OR ajustada 1,16; IC95% 1,06; 1,27) apresentaram maiores chances de ter um filho com cardiopatia congênita. As mães em união estável apresentaram maior chance (OR ajustada 1,44; IC95% 1,37; 1,53), enquanto para mães divorciadas a associação não se manteve significativa após ajuste.

As gestações pré-termo apresentaram chances 2,6 vezes maiores de ocorrência de cardiopatias congênitas em comparação ao grupo de referência (OR ajustada 2,61; IC95% 2,45; 2,78). No modelo inicial, menos de 7 consultas pré-natais estiveram associadas ao aumento de chances de ocorrência de cardiopatias congênitas (OR bruta 1,20; IC95% 1,14; 1,26), porém essa associação não se manteve após ajuste. Gestações duplas apresentaram maiores chances em comparação às únicas (OR ajustada 1,14; IC95% 1,00; 1,30). As chances foram mais que duplicadas (OR ajustada 2,60; IC95% 1,57; 4,31) nas gestações triplas ou mais. Mães residentes em áreas previamente identificadas como agrupamentos espaciais de risco apresentaram quase o triplo de chances de ocorrência de cardiopatias congênitas em relação às residentes fora dessas áreas (OR ajustada 2,97; IC95% 2,82; 3,12).

## Discussão 

Os achados deste estudo indicaram que tanto características maternas e obstétricas quanto fatores territoriais estão associados à ocorrência de cardiopatias congênitas no município de São Paulo. Observaram-se maiores chances entre mães com idade materna avançada, baixa escolaridade, de raça/cor da pele amarela e preta, em união estável, com gestações pré-termo e múltiplas, e residentes em áreas de agrupamentos espaciais de risco.

A análise espacial evidenciou padrão geográfico que indica desigualdades na distribuição do risco de cardiopatias congênitas no município de São Paulo. Destacou-se uma área de risco localizada em uma região com elevada proporção de domicílios em favelas e comunidades urbanas e concentração de população de baixa renda (5). Esse achado reforçou evidências de que condições de vulnerabilidade social e ambiental estão associadas a maiores riscos à saúde, incluindo a ocorrência de anomalias congênitas (7,18). 

As características maternas e obstétricas se mostraram relevantes para compreender os padrões de ocorrência dessas malformações. A idade materna avançada foi um dos fatores associados, em consonância com estudos prévios que relacionam gestações tardias ao aumento de alterações cromossômicas, como a síndrome de Down (19). A associação entre menor escolaridade e maiores chances, embora de magnitude moderada, reforça o papel da escolaridade como marcador do contexto socioeconômico, refletindo desigualdades no acesso à informação, à qualidade do pré-natal e aos serviços de saúde (20,21). 

A análise da variável raça/cor da pele revelou que mães amarelas e pretas apresentaram maiores chances de ocorrência de cardiopatias congênitas, em comparação às mães brancas, porém de menor magnitude entre as mães pretas. Pesquisas em diferentes contextos apontaram resultados semelhantes. Em populações asiáticas, houve maior prevalência dessas anomalias na população amarela, possivelmente relacionada a fatores genéticos e ambientais específicos (2). No contexto brasileiro, investigações sugeriram que mães pretas estão mais expostas a vulnerabilidades sociais, como baixa escolaridade e menor número de consultas pré-natais, condições frequentemente associadas a piores desfechos perinatais (22). 

Neste achado, o estado civil materno também esteve associado à ocorrência de cardiopatias congênitas, com maiores chances entre mães em união estável, em comparação às solteiras. Não houve, contudo, evidências suficientes para sustentar uma relação causal direta, sendo possível que essa variável atue como marcador indireto de características contextuais, como perfil demográfico ou padrões de acesso aos serviços de saúde. A interpretação desse achado requer cautela e deve ser aprofundada em estudos que incorporem informações adicionais sobre o contexto social e familiar.

Entre os fatores obstétricos analisados, a prematuridade destacou-se, apresentando uma das maiores chances associadas, corroborando resultados de pesquisas que apontaram o parto pré-termo como importante fator de risco para malformações cardíacas (23). Esse efeito pode estar relacionado a interferências no desenvolvimento fetal em períodos críticos da gestação, aumentando a vulnerabilidade a defeitos estruturais (23).

Neste estudo, o número insuficiente de consultas pré-natais não apresentou associação com o desfecho após ajuste do modelo. Apesar disso, evidências indicaram que o acompanhamento inadequado durante a gestação está associado ao maior risco de malformações congênitas e outros desfechos neonatais adversos (24). No Brasil, o Ministério da Saúde recomenda a realização de, no mínimo, seis consultas durante a gestação, consideradas essenciais para a redução da morbimortalidade materno-infantil e para a detecção precoce de agravos (25).

As gestações múltiplas, especialmente as de ordem superior, também se associaram às maiores chances de ocorrência de cardiopatias congênitas, achado consistente com a literatura, que apontou maior vulnerabilidade e complexidade no acompanhamento desses casos (26). Esse resultado evidenciou a importância de estratégias de pré-natal intensificadas e de acompanhamento especializado para gestações múltiplas, com vistas à prevenção e à detecção precoce dessas malformações.

A área de residência materna mostrou-se importante fator associado à ocorrência de cardiopatias congênitas, com maiores chances em áreas identificadas como agrupamentos espaciais de risco. Esse achado reforça que o território não é apenas o cenário onde os eventos ocorrem, mas também é um determinante ativo para os desfechos.

A utilização da variável “residência em agrupamento” como proxy territorial permitiu integrar a dimensão espacial à análise individual. Embora este estudo não tenha incorporado indicadores específicos sobre o ambiente social ou físico das áreas de residência, evidências indicaram que exposições como à poluição atmosférica e a agentes químicos e barreiras no acesso a serviços de saúde estão associadas à ocorrência de desfechos adversos à saúde, incluindo anomalias congênitas (7,8). A associação observada entre área de residência e cardiopatias congênitas pode estar refletindo o efeito acumulado de exposições contextuais.

Embora amplamente aplicada em diferentes áreas da saúde pública, a análise espacial ainda é pouco explorada no estudo das cardiopatias congênitas. Essa abordagem tem se mostrado promissora na identificação de padrões geográficos de risco e na formulação de hipóteses sobre determinantes territoriais do agravo (27,28). Em grandes centros urbanos, como o município de São Paulo, seu uso pode representar uma ferramenta estratégica para subsidiar ações de vigilância e planejamento de ações em saúde.

Entre as limitações deste estudo, destacou-se a impossibilidade de geocodificação por endereço, restringindo a análise espacial ao nível dos distritos administrativos. Embora essa unidade permita captar padrões relevantes, sua escala pode ocultar heterogeneidades internas significativas, sobretudo em distritos extensos e densamente povoados. 

Outra limitação referiu-se à fonte de dados utilizada. Embora o Sistema de Informações sobre Nascidos Vivos seja amplamente empregado em pesquisas epidemiológicas, trata-se de registro secundário que pode apresentar subnotificação de casos leves e restrições na coleta de informações contextuais, além de não contemplar diagnósticos estabelecidos tardiamente. Tais lacunas podem introduzir imprecisões nas análises e dificultar a inclusão de variáveis explicativas relacionadas ao contexto ambiental, reduzindo a sensibilidade da análise. 

A utilização de uma variável territorial agregada (residência em agrupamento espacial) como proxy do contexto espacial não substituiu o uso direto de indicadores ambientais ou socioeconômicos. A ausência desses indicadores nas análises limitou o controle de possíveis fatores de confusão relacionados ao território, o que representou limitação metodológica do estudo. Estudos futuros poderão aprofundar essa abordagem ao integrar dados ambientais mais detalhados e incorporar o componente espacial diretamente à estrutura dos modelos analíticos. Isso permitirá identificar padrões de risco não explicados apenas por características individuais, ampliando a compreensão dos determinantes das cardiopatias congênitas.

Por fim, destacou-se uma limitação inerente ao delineamento adotado. Por se tratar de estudo transversal, os achados não permitiram inferência de causalidade, sendo necessário cautela ao discutir possíveis implicações para políticas públicas ou ações em saúde.

Os resultados deste estudo indicaram que as características sociodemográficas maternas, os fatores obstétricos e o território de residência estão associados à ocorrência de cardiopatias congênitas no município de São Paulo. Ao evidenciar perfis maternos vulneráveis e distribuição territorial desigual do risco, o estudo ofereceu subsídios para a vigilância de anomalias congênitas e para o planejamento de ações prioritárias em saúde pública, no âmbito do Sistema Único de Saúde, voltados à prevenção, à detecção precoce e ao cuidado das cardiopatias congênitas. Tais evidências são especialmente relevantes para reduzir a morbimortalidade associada a essas condições e qualificar as ações voltadas à saúde materno-infantil.
